# Effects of anti-retroviral therapy on baseline serum interleukin-18 levels in HIV–I infected patients relative to viral suppression and CD4+ gain: A prospective pilot study

**DOI:** 10.37796/2211-8039.1406

**Published:** 2023-06-01

**Authors:** Olayemi Balogun, Bukhari I. Shuaib, Abdulrasheed Usman, Aminu A. Yusuf, Bolanle O.P. Musa, Obiako O. Reginald, Aliyu A. Babadoko

**Affiliations:** a Department of Medical Microbiology, Ahmadu Bello University Teaching Hospital, Zaria, Nigeria; b Anti-Retroviral Therapy (ART) Laboratory, Ahmadu Bello University Teaching Hospital, Zaria, Nigeria; c Department of Pharmacology and Therapeutics, Faculty of Pharmaceutical Science Ahmadu Bello University, Zaria, Nigeria; d Department of Haematology, Faculty of Clinical Sciences, Bayero University Kano, Nigeria; e Department of Haematology and Blood Transfusion, Aminu Kano Teaching Hospital, Nigeria

**Keywords:** Human immunodeficiency virus, cART, HIV-1 RNA, Interleukin-18, Interquartile range

## Abstract

**Background:**

In HIV infection, dysregulation of cytokines, including interleukin 18 (IL-18), has been linked to poor clinical outcomes in studies mainly conducted in resource-rich countries. This phenomenon has not been well-studied in resource-limited settings where outcomes could be confounded by exposure to endemic infections and genetic factors.

**Objectives:**

Therefore, the influence of immunological and virological status of HIV-infected, antiretroviral therapy (ART)-naïve patients on serum IL-18 levels at baseline (pretreatment) and 24 weeks following initiation of combination ART (cART24) in a resource-limited setting was investigated.

**Methods:**

Using the cross-sectional and longitudinal mixed method design, a total of Forty-four (44) newly diagnosed consenting HIV patients were consecutively recruited during routine clinic visits at the Nasara Treatment & Care Centre of the Ahmadu Bello University Teaching Hospital (ABUTH), Zaria, Nigeria between December 2016 to January 2018, and followed up for 24 weeks on initiation of first-line cART.

**Results:**

Serum IL-18 concentrations, CD4+ T-cell counts (CD4+) counts, and HIV1 RNA levels *were determined at baseline and cART24. There was little CD4*+ *count gain in both* <200 and ≥ 200 cell/mm^3^subgroups despite the high proportion of subjects having virological suppression (n = 35, [80%]) at cART24. However, at cART24 there was a more than a threefold decrease in the level of IL-18 concentration compared to baseline in patients with <200 cells/mm^3^ and a significant decrease in the median plasma IL-18 concentration in patients with HIV1 RNA <1000 cp/mL at cART24. A multivariate logistic regression model shows IL-18 intermediate quartile to be more related to immunological poor gain as compared to the highest quartile.

**Conclusion:**

Our study found high baseline and significantly low levels of IL-18 at cART24 in virologically suppressed subjects but not among virological non-suppressed responders despite comparable IL-18 levels by CD4+ T cell count strata at cART24. These findings have implications for risk stratification and treatment outcomes in HIV-positive persons.

## 1. Introduction

Significant disparities in the burden of HIV/AIDS exist worldwide, with economically disadvantaged communities bearing a disproportionate burden of the disease. Sub-Saharan Africa (SSA) alone accounts for approximately 60% of the daily HIV infection worldwide [1]. In low-income settings, HIV-positive persons frequently present with late-stage disease and a very low CD4 cell count [2,3]. Late diagnosis; here defined as a first-reported CD4 count <200 cells/μL, remains a major barrier to starting early antiretroviral treatment at a high CD4+ T-cell count in low-income countries [1,2]. In one study, the median CD4 cell count in subjects initiating highly active antiretroviral therapy (HAART) was ~250 cells/mm^3^ in low-resource settings, but close to 500 cells/mm^3^ in resource-rich countries [2]. Despite the initiation of HAART, most patients who presented late suffer from opportunistic infections (OI) and premature death despite achieving viral suppression [3,4]. The most frequently encounter OI’s includes Bacterial pneumonia, Candida infection, Tuberculosis, and Infectious diarrhea [1,6]. It is estimated that 90% of HIV-related deaths are caused by opportunistic infections, compared to 7% due to cancers and 3% from other related causes [2]. A multitude of interacting factors influences the progression of HIV infection to AIDS [7]. One such factor is activated inflammation, which has been linked to adverse outcomes in HIV including early progression to AIDS [4,8,11–13].

Cytokines are crucial to cellular signaling pathways and intercellular communication, with pleiotropic effects in various organs, and thus play a critical role in modulating the immune response [13,17]. Induced inflammatory immune activation is linked to poor outcomes in chronic HIV infection due to its association with CD4+ T cell count depletion, higher viral replication, and increased risk of non-immunological complications including cardiovascular diseases and kidney dysfunction [14,16,20]. Cytokines exert unique immunologic and biological effects, playing a multifunctional role as proviral and antiviral factors in disease pathogenesis. Serum markers of inflammation in HIV infection have been identified, including Interleukin 18 (IL-18), a proinflammatory, proapoptotic and proatherogenic cytokine belonging to the interleukin-1 family of cytokines [22,25,28].

In general, immune responses enhance the capacity of the host’s cellular immunity, particularly against viral infections [33]. However, dysregulation of some cytokines, including IL-18, may lead to a disproportionate immune activation [15,23], decreased survival of cells, and promotion of various immune and non-immune cell deaths [23]. Excessive IL-18 action in HIV infection has also been linked to the development of HIV-associated lipodystrophy [20,24].

The role of cytokines in immuno-virological pathology in HIV infection is relatively well documented in developed countries, environmental exposure to endemic pathogens may affect the basal repertoire of inflammatory status and induce a cytokine storm that drives unique pathologies in resource-limited settings [18,35]. There is currently limited clinical data on the role of IL-18 during HIV-1 infection in resource-limited settings. The aim of this study was, therefore, to determine the serum levels of IL-18 in responses to cART based on HIV1 RNA level and CD4+ T cell counts at baseline and 24 weeks after the commencement of cART in HIV1 infected persons living in a resource-limited setting.

## 2. Method

### 2.1. Study settings and design

This was a pilot prospective study that evaluated serum IL-18 levels at baseline and cART24 in newly diagnosed HIV-positive patients without previous antiretroviral medication and compared changes in their CD4+ counts and plasma viral loads over the study period. The study was carried out at Nasara Treatment & Care Centre of the Ahmadu Bello University Teaching Hospital (ABUTH), Zaria, Nigeria, which is a U.S. President’s Emergency Plan for AIDS Relief (PEPFAR)-supported HIV treatment facility. The study was conducted in December 2016 and January 2018 followed up for 24 weeks on the initiation of first-line cART. This centre has been cumulatively responsible for screening and providing treatment as well as ART adherence counseling to PLWH.

### 2.2. Study participants

The participants included confirmed HIV-positive adults (aged ≥18 years) who were ART-naïve at baseline. ART-experienced patients, sex workers, and pregnant women were excluded. Patients with apparent co-morbidities were also excluded. Eligible participants were consecutively selected and followed up for 6 months (24 weeks). All participants were on a first-line cART regimen for 24 weeks in line with the “test and treat” WHO strategy [1,2]; a fixed dose combination of Efavirenz/Lamivudine/Tenofovir combined in a single pill.

### 2.3. Ethics statement and informed consent

The study protocol was approved by the research ethics committee of the ABUTH, Zaria, Nigeria (ethics approval number: ABUTH/HREC/515/2015). All study procedures were conducted per the Declaration of Helsinki. Written informed consent was obtained from participants before participation; voluntariness and strict confidentiality were maintained throughout the study.

### 2.4. Socio-demographic and clinical data

Relevant socio-demographic characteristics and clinical data (Table 1) were obtained using a structured questionnaire and a review of the patient’s clinical records.

### 2.5. IL-18 measurement

IL-18 was assayed using Quantikine, an antigen-based, solid phase, sandwich Enzyme-Linked Immunosorbent Assay (ELISA) for the Human IL-18 BPa. The assay was performed according to the manufacturer’s instructions. The kit has a sensitivity of 7.52 pg/ml and a specificity of 10.0 pg/ml.

### 2.6. Plasma HIV–I viral load

The plasma HIV-1 RNA was determined using the COBAS® Ampliprep/COBAS® Taqman® HIV-1 Test, v2.0. (Roche Diagnostics, Indianapolis, USA). Specimen preparation and assay were all performed according to the manufacturer’s instructions. The dynamic range was from 20 to 10.000.000 copies/mL.

### 2.7. Determination of absolute CD4+ T-cell count

Clusters of differentiation (CD) 4+cell count in whole blood were determined using Partec™. Cyflow analyzer (Model SL3, Germany) based on manufacturer’s instructions.

### 2.8. Definitions

CD4+ decline was stratified by CD4+ T-lymphocyte count (<200 and ≥200 cells/mm^3^). HIV viral load <1000 copies/mL was considered virologically suppressed, while ≥1000 copies/mL were considered virologically non-suppressed. Low CD4+ recovery following cART initiation was defined as CD4+ increase <50 cells/mm^3^ at cART24 despite HIV RNA <1000 copies/mL [5,7,30].

### 2.9. Statistical analyses

Data generated were coded using a Microsoft Excel spreadsheet and soft copies were kept in a password-protected computer. Statistical analyses were performed in software R v. 4.0.3. Data were cleaned and tested for normality using the Shapiro–Wilks normality check. Data were then expressed as median (interquartile range, IQR), or percentage (frequency) after failing normality checks. Interleukin 18 levels were parameterized into quartiles (Q1: 25th Percentile, Q2: 50th Percentile, and Q3: 75th Percentile) and their median values were used to represent each quartile. Following the longitudinal and cross-sectional research design, data were compared using Wilcoxon rank-sum test also known as Wilcoxon unpaired two-sample test statistic (cross-sectional component) and the Wilcoxon matched-pair signed rank test to analyze the longitudinal component. Multiple comparisons of ranks were followed by the Bonferroni correction test which allows the adjusting of P-values to correct for the occurrence of false positives. The level of significance was set at a 95% confidence interval (CI), assuming p ≤ 0.05.

To evaluate the response to cART, the proportion of patients’ IL-18 levels achieving a plasma HIV viral load (below and above 1000 copies/ml) and the absolute CD4+ cell count (below and above 200 cell/mm^3^) at baseline and 24 weeks (ART24) were computed. The Chi–Square test was used to determine statistical association. To account for potential confounders, a multivariate logistic regression model was used to control for the biologically plausible variables included in our study design: age, sex, baseline BMI, baseline WHO clinical, and baseline HIV RNA level. In this model, baseline IL-18 quartiles were used as determinants, and CD4+ counts at cART24 as the response variable. The robust standard errors for the parameter estimates were adopted to control for mild violation of homoscedasticity. We use the R package sandwich to obtain the robust standard errors and calculated the p-values accordingly. Together with the p-values, a 95% confidence interval using the parameter estimates and their robust standard errors was calculated. p-value was set as p ≤ 0.05.

## 3. Results

The demographic and clinical characteristics of the participants are shown in Table 1.

### 3.1. Longitudinal subgroup analysis of IL-18, CD4 counts, and HIV RNA load at cART24

**Fig. 1:**
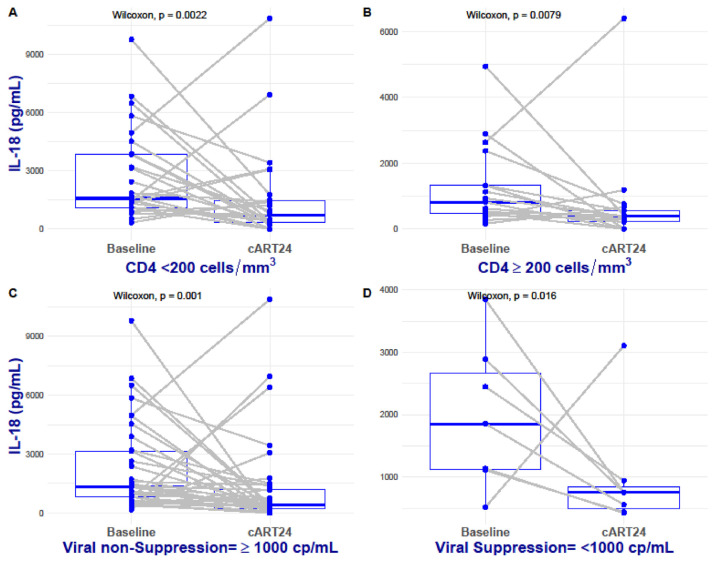
Effect of cART following 24 weeks of treatment on interleukin 18 levels along their immunological and virological subgroup (longitudinal). Dots represent each subject followed up along the predefine immunological and virological stages. A and B represent the CD4+ strata; C and D represent the Viral Load strata; Wilcoxon rank sum paired-ranked test (P ≤ 0.05).

Patients in both the immunological and HIV viral load strata had their IL-18 levels significantly suppressed at cART24 (p < 0.01, Fig. 1). The median HIV viral loads were significantly lower at cART24 compared to their baseline levels irrespective of their stratifications (p < 0.05), and interestingly, all subjects had a median viral load <200 cp/mL (≥1000 at cART24: 129 [IQR: 20–550] cp/mL and; <1000 at cART24: 49 [IQR: 37–88] cp/mL; Table 2). While cART24 induced a marked decline in IL-18, immunological and virological treatment failure was associated with persistently raised IL-18 levels (Fig. 1A and C).

### 3.2. Cross-sectional subgroup analysis of IL-18, CD4 counts, and HIV RNA load at cART24

**Fig. 2:**
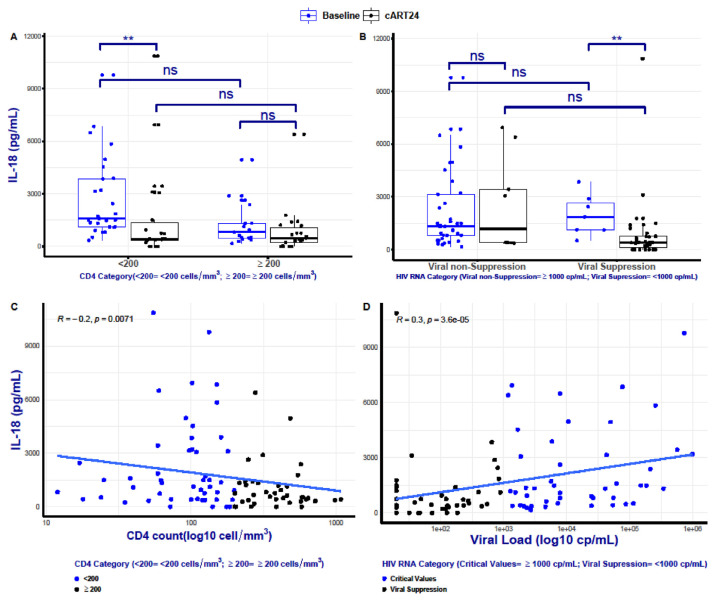
Subgroup comparison and correlation of interleukin 18 along predefine immunological and virological stages (cross-sectional component). Median comparisons of interleukin serum levels along various immunological CD4 cell populations and virological responses following cART (A and B). Correlation between plasma IL-18 concentrations and various stages of HIV viral loads and CD4 cell count in the HIV-infected population studied (C and D). The interquartile ranges (boxes), medians (bars within boxes), and entire ranges (vertical bars) are shown for all patient groups. Each black and blue dots represent individual participants. *P ≤ 0.05; **P ≤ 0.01.

Serum IL-18 level was significantly higher at baseline in individuals with a CD4+count <200 cells/mm^3^ compared to cART24 (median: 1589[IQR: 1114–3866] pg/mL vs 419[IQR, 362–1363]pg/mL, P < 0.01). Among individuals with CD4+ count ≥200 cells/mm^3^ at baseline, there was an approximately 2-fold reduction at cART24 (median: 815 [IQR, 479–1328] pg/mL vs 448[IQR, 235–1080]pg/ml, P < 0.05; Fig. 2A). In parallel, virologically suppressed individuals also had a statistically significant decrease in the median serum IL-18 concentration at cART24 (1856[IQR, 1126–2670] pg/mL vs 413[IQR, 111–758] pg/mL, P < 0.001; Fig. 2B). However, the virologically non-suppressed group had a non-significant decrease in their median IL-18 level at cART24 compared to baseline (1328[808–3146] pg/mL vs 1186[387–3432] pg/mL, P > 0.05; Fig. 2B). Table 3 shows an increase in the number of patients who had their viral load suppressed at cART24 (n=35 [80%]). There was a non-significant CD4+ count increment among the group with baseline CD4+ count <200 cell/mm^3^ (Table 3). There was also no significant change observed in the group with CD4+ counts ≥200 cells/mm^3^ (Table 3).

### 3.3. Changes in IL-18 levels at baseline and cART24

Serum IL-18 levels were statistically different along the immunological and virological categories (Table 4). However, participants with CD4+ T cell counts <200 cells/mm^3^ and ≥1000 cp/mL had more elevated levels of the IL-18 (Q3) at both time points (baseline: 10 [37%] and; cART24: 4 [18%] by immunological status; baseline: 10 [27%] and; cART24: 3 [33%] by virological HIV RNA status; Table 4). Virologically suppressed participants at cART24 had more reduction in serum IL-18 levels compared to the virologically non-suppressed group (Q1: 32 [91%] vs. 5 [56%]; Table 4). In contrast, the participants with a CD4+ count of <200 cells/mm^3^ had a comparable proportion of subjects having IL-18 levels reduced at cART24 (17 [77%] vs. 20 [90%]; Table 4). A multivariable logistic regression model showed that IL-18 levels were significantly associated with immunological outcomes (Table 5). The second baseline interleukin-18 quartiles had the highest odd ratio (minimum–maximum: 1722–3063 pg/mL, Table 5) while the first quartile had a negative odd ratio.

### 3.4. Association of HIV RNA and CD4+ counts with serum IL-18 levels at baseline and at cART24

Kendall’s linear regression model was used to measure the extent of viral load and CD4+ count association with serum levels of IL-18 (Fig. 2). Irrespective of the immunological or virological status, IL-18 values were negatively associated with CD4+ counts (Kendall’s tau = −0.2, P = 0.007; Fig. 2C) and positively with HIV viral loads (tau = 0.3, P < 0.001; Fig. 2D).

## 4. Discussion

Findings from this study showed a high prevalence of poor CD4+ T cell recovery following the commencement of cART after adjusting for the CDC immunological stage despite virologic suppression. The high proportion of patients presenting with low baseline CD4+ T cell count could be responsible for the poor immune response observed. Although CD4+ T cell count and HIV viral load are routinely monitored in HIV/AIDS patients, there are varying reports regarding the clinical relevance of CD4+ T cell counts [27]. However, continual loss of CD4+ T lymphocytes drives clinical progression to acquired AIDS [21]. Therefore, the paradoxical VL suppression with low CD4 gains following cART24 in this study could represent a detrimental clinical sequela. This preliminary inference is supported by findings by Han et al. [8], that showed poor CD4+ cell gain following ART initiation for up to 32 weeks persisted through 5 years of follow-up.

Available literature has reported the percentage of patients with a low CD4+ count recovery to vary between 15% and 30% [11] and 10%–40% [10] depending on the definition used and duration of initiating cART. Results from our study show a significant number of patients did not achieve immunological restoration despite virological suppression after a period of 24 weeks of cART. Reports from the literature reveal low-level immune activation as measured by some pro-inflammatory cytokines to be associated with poor immune response [19]. However, dampening residual immune activation by pharmacologically inactivating these immune pathways could also help improve immune function in HIV-infected individuals [9]. A brief review of the literature characterizes HIV infection into acute or primary phase and ends with a symptomatic phase associated with an intense CD4+ T lymphocyte depletion and a progressive increase in the serum HIV RNA viral load (VL) [21,22]. These phases are associated with immune activation which manifests in the elevation of numerous plasma cytokines and chemokines [16,19,21,31]. However, unlike the viremia cycle, cytokine levels drop to an intermediate level which runs in parallel to the chronic phase of HIV disease [26]. The finding from our study might be suggestive of a potential role of IL-18 in the pathogenesis of HIV infection. High baseline and significantly low levels of IL-18 at cART24 were observed in virologically suppressed subjects. In contrast, IL-18 was only mildly reduced at cART24 in virological non-responders despite having a much lower IL-18 concentration at baseline. This may be reflective of a multitude of interacting factors including the phase which is most responsive to treatment. Some studies have reported early initiation of cART in HIV infection to reduce the viral reservoir and diminish HIV transmission [4,30]. However, other studies argue that during the early stages of HIV-1 infection, characterized by cytokine storm, IL–18 might suppress HIV-1 by increasing the T lymphocytes’ type 1 (Th1) immune response and reducing CXCR4 co-receptor expression [24]. Early initiation of cART has been reported to abrogate HIV-induced cytokine storms during the acute phase of infection [7,13]. Additionally, our study found higher HIV RNA load to correlate with higher IL-18 levels. This association between HIV RNA suppression and serum IL-18 is consistent with findings that showed IL-18 involvement in HIV pathogenesis [20,29]. Nevertheless, the biology of IL-18 could be complicated by interactions between the inflammatory response and viral replication. Our findings support repeated measure of IL-18 in cART-naïve persons for early identify patients that would not responds to CD4 gains despite virological suppression to prevent clinical progression to AIDs and non-AID events.

Early observations have shown that the selective depletion of the CD4+ T cells is accompanied by aberrant immune activation of all the components of the immune system in patients with HIV infection [13]. Although cART has been shown to significantly suppress the level of proinflammatory cytokines including IL-18, there remains evidence of persistent immune activation, albeit at a low level [16]. The clinical significance of persistent immune activation in spite of treatment is suggested by the increased risks of HIV and non-HIV events associated with elevated levels of pro-inflammatory cytokines [23,32]. In agreement with this observation, our findings show significantly low levels of proinflammatory IL-18 cytokine at CD4+ T cell counts <200 cell/mm^3^, with only ~2-fold decrease in IL-18 level, observed in patients with ≥200 cell/mm^3^ CD4+ T cell counts at baseline (Fig. 2A). Despite these differences, a comparable IL-18 level in both CD4+ T cell counts strata at cART24 was observed (Fig. 2B). This observation may be suggestive of a preserved immunity. However, to achieve a CD4+ gain of more than 50 cells/mm^3^ in these subjects, the authors posit that further IL-18 suppression may be needed to achieve this gain. Persistent elevation of IL-18 was also observed in patients that did not achieve immunological gain (CD4) and virological suppression at cART24. This finding is consistent with previous studies [14,23] that show persistent IL-18 elevation to be associated with non-AIDs events such as cardiovascular disease. Therefore, a well-controlled study will be required to further elucidate the clinical relevance of such modulatory action.

There is a growing body of evidence [37] that markers of inflammation may have ethnic and genetic differences [37,38]. Such differences are also seen in the association with inflammatory markers to HIV/AIDS progression. The findings from our study reveal a much higher interleukin level following *in situ* data analysis (baseline) when compared with similar cohort studies in Zambia–North America and Baltimore, Maryland (29, 31, 32). Although the interleukin-18 levels were of subjects with high viral load and reduced CD4 count, they did not use similar stratification. Racial and genetic diversity, along with social and environmental factors may contribute to the varying association of IL-18 levels which is also seen in many settings [34,37,38]. To support this proposition, Negi *et al* [35] reported higher serum IL-18 levels in African Americans compared with their white counterparts. However, the exact mechanism of the immunomodulatory effect of IL-18 on CD4+ gains needs further studies.

In contrast to previous studies that consistently used baseline upper quartile (Q3: 75th quartile) as predictors of immunological failure and clinical progression of HIV infection, our study shows, for the first time, that an intermediate quartile (Q2: 50th percentile) was a stronger predictor of immunological failure in our study population. This observation is further strengthened by the lack of cART suppression of baseline IL-18 level despite lower baseline median IL-18 level in virologically non-suppressed subjects (>1000 cp/mL) as compared to virologically suppressed subjects. However, baseline IL-18 concentration within the 25th quartile was suggestive of a preserved immune function. This variability may reflect heterogeneous virus-host dynamics and cART treatment status, including socioeconomic variability across geographies, the use of appropriate normative data, and the handling of confounders [37,38]. Furthermore, these factors may determine the basal inflammatory state due to their interactions and subsequent exposure to other endemic pathogens [36,38].

Our study also found a positive correlation between serum IL-18 and HIV-1 viral, a negative correlation between serum IL-18 and CD4+ T cell count, and decreased serum IL-18 concentrations following effective anti-retroviral therapy. These findings are consistent with other studies investigating the clinical significance of IL-18 suppression as a measure of cART response and monitoring of HIV progression [4,33].

Some of the limitations of our study include the relatively small sample size and short follow-up period which limits the generalizability of our findings. Laboratory screening for concomitant infections other than HIV-1 was not carried out. Also, ART adherence among study participants was not analyzed. This could potentially affect the determination of serum IL-18 levels. Despite these limitations, our study provides useful findings of clinical relevance that can be built upon. A scale-up to involve a larger sample size, as well as mechanistic investigations, would be needed to further elucidate the clinical relevance of the trend observed in this study.

## 5. Conclusion

Our study highlights some of the dynamics of IL-18 levels at various stages of CD4+ T cell counts and HIV RNA status in HIV-positive patients at baseline and at 24 weeks after commencing cART. Our findings have relevance for early identification and stratification of patients at risk of virological or immunological failure (non-response), with implications on treatment outcomes in HIV-positive persons. Future studies should focus on therapeutic strategies focused on not only viral suppression but immunological reconstitution of HIV-infected patients to avoid clinical progression to AIDS. Further reduction of IL-18 to a comparable level in healthy subjects should be explored as a pharmacological target for the achievement of such immunological reconstitution.

## Data availability statement

The data sets used and/or analyzed during the current study are available from the corresponding author upon reasonable request.

## Figures and Tables

**Table 1:** Baseline demographic and clinical characteristics.

Variables	All participants n = 44
Age, mean (±SD)	38.23 (±9.3)
Gender n (%)	
Male	14 (31.8)
Female	30 (68.2)
Last WHO clinical stage before initiating ART	
1 and 2	36 (81.8)
3 and 4	8 (18.2)
Body mass Index (Kg/m^2^,%)	
<18.5	9 (20.5)
18.5–24.9	28 (63.6)
25–29.9	6 (13.6)
30–39.9	1 (2.3)
Viral load (copies/ml, %)	
<1000	7 (15.9)
1000–10,000	37 (84.1)

**Table 2:** Longitudinal cellular response and HIV RNA Load following 24 Weeks on ART (cART24).

	Baseline			cART24			a*P* Value
	
	Median	IQR	n^1^	Median	IQR	n^2^	
CD4 counts, cells/mm^3^							
≥200	386	273–484	17	388	270–579	17	0.623
<200	101	55–134.5	27	125	105.5–200.5	27	<0.001
Viral load, cp/mL							
≥1000	10,731	4789–76833	37	129	20–550	37	<0.001
<1000	724	527–825	7	49	37–88	7	0.016

a Wilcoxon matched-pair signed rank test p-value ≤0.05.

**Table 3:** Cross-sectional cellular Immune response and HIV RNA Load at Baseline and at 24 Weeks on ART (cART24).

	Baseline			cART24			aP-Value
	
	Median	IQR	n^1^	Median	IQR	n^2^	
CD4 count, cells/mm^3^							
<200	101	55–135	27	114	73–137	22	0.35
≥200	386	273–484	17	384	262–567	22	0.955
Viral Load, cp/mL							
≥1000	10,731	4789–76833	37	1850	1367–2105	9	0.0036
<1000	724	527–825	7	49	20–133	35	0.0001
IL-18 Quartiles (Q) (pg/mL)							
Q1	913	518–1325	27	397	218–727	37	0.0004
Q2	2413	1987–2587	6	2419	2096–2741	2	0.857
Q3	4947	3866–6170	11	6395	3432–6937	5	0.743

n^1^: the number of at baseline; n^2^: the number of patients’ responses at 24 weeks on cART; cART24: 24 weeks on combination antiretroviral Therapy; Quartile (Q): Q1 (25th percentile); Q2 (50th percentile); Q3 (75th percentile); IQR: Interquartile Range.

a Wilcoxon rank-sum test (also known as Wilcoxon unpaired two-sample test statistic).

**Table 4:** Comparison between the different quartiles of IL-18 levels and different levels of CD4+ counts (<200 and ≥ 200) and HIV RNA load (virally suppressed and virally non-suppressed) at baseline and 24 weeks on cART.

Variables		Variable category	aIL-18 Quartiles (Q), n (%)				bP-Value

			Q1	Q2	Q3	Total	
CD4 T-cell Count (cells/mm3)							0.03
	Baseline	<200	14 (52)	3 (11)	10 (37)	27	
		**≥200**	13 (76)	3 (18)	1 (6)	17	
	ART24	<200	17 (77)	1 (5)	4 (18)	22	
		**≥200**	20 (90)	1 (5)	1 (5)	22	
HIV RNA Load (copies/mL)							0.004
	Baseline	≥1000	24 (65)	3 (8)	10 (27)	37	
		<1000	3 (43)	3 (43)	1 (14)	7	
	ART24	≥1000	5 (56)	1 (11)	3 (33)	9	
		<1000	32 (91)	1 (3)	2 (6)	35	

a Continued immune activation was defined for each subgroup biomarker separately based on the distribution at baseline (pre-ART). Q1: 898; Q2: 1607; Q3: 3082.

b Chi-square test.

**Table 5:** Multivariable logistic regression analysis for immunological failure at 24 Weeks (ART24).

IL-18 pg/ml (Median)	Range (min–max)	aOR, 95% CI	P Value
Q1: 913	168–1589	−0.21 (−0.30, −0.11)	<0.001
Q2: 2413	1722–3063	0.35 (0.19, 0.51)	<0.001
Q3: 4947	3106–10861	0.14 (0.07, 0.20)	<0.001

Abbreviations: CI, confidence interval; IL, interleukin; aOR, adjusted odds ratio.

a Model was adjusted for age, sex, baseline BMI, baseline WHO clinical stage, and baseline HIV viral load. Quartile (Q): Q1 (25th percentile), Q2 (50th percentile), Q3 (75th percentile).

## Abbreviations

ABUTH: Ahmadu Bello University Teaching Hospital
AIDs: acquired immunodeficiency syndrome
ART: antiretroviral therapy
cART: combination antiretroviral therapy
cART24: combination antiretroviral therapy at 24 weeks
CD4: cluster of differentiation 4
ELISA: Enzyme-Linked Immunosorbent Assay
HAART: highly active antiretroviral therapy
HIV: human immunodeficiency virus
HIV-1 RNA: human immunodeficiency virus 1 ribonucleic acid
IL-18: Interleukin 18
IQR: interquartile range
pg/ml: Picograms per milliliter
QS: Quantitation Standard
SSA: Sub Saharan Africa
